# Study on Purification and Characterization of Polyphenol Oxidase from *Acetes chinensis*

**DOI:** 10.3390/molecules26247545

**Published:** 2021-12-13

**Authors:** Jianyou Zhang, Guangcheng Zhou, Lifeng Fei, Lifan Chen, Lei Sun, Fei Lyu, Yuting Ding

**Affiliations:** 1 College of Food Science and Technology, Zhejiang University of Technology, Hangzhou 310014, China; zhjianyou@zjut.edu.cn (J.Z.); zhougc1997@163.com (G.Z.); Feilifeng1997@163.com (L.F.); zjutcailiaokeji@163.com (L.C.); sl20887@163.com (L.S.); dingyt@zjut.edu.cn (Y.D.); 2 Key Laboratory of Marine Fishery Resources Exploitment & Utilization of Zhejiang Province, Hangzhou 310014, China; 3 National R&D Branch Center for Pelagic Aquatic Products Processing (Hangzhou), Hangzhou 310014, China; 4 Collaborative Innovation Center of Seafood Deep Processing, Dalian Polytechnic University, Dalian 116034, China

**Keywords:** *Acetes chinensis*, polyphenol oxidase, purification, characterization

## Abstract

*Acetes chinensis* (belonging to the Decapoda Sergestidae genus) is widely distributed in East Asian waters and is extremely widespread and present in the shallow coastal areas of China. Polyphenol oxidase (PPO), which was extracted from *Acetes chinensis*, was purified in a four-step procedure involving phosphate-buffered saline treatment, ammonium sulphate precipitation, DEAE-Cellulose chromatography, and Phenyl-Sepharose HP chromatography, and then, its biochemical characterization was measured. The specific activity of the purified enzyme was increased to 643.4 U/mg, which is a 30.35 times increase in purification, and the recovery rate was 17.9%. L-dopa was used as the substrate, the enzymatic reactions catalyzed by PPO conformed to the Michaelis equation, the maximum reaction velocity was 769.23 U/mL, and the Michaelis constant K_m_ was 0.846 mmol/L. The optimal pH of PPO from *Acetes chinensis* was 7.5, and the optimal temperature was 35 °C. The metal ions experiment showed that Mn^2+^ and K^+^ could enhance the activity of PPO; that Ba^2+^ and Ca^2+^ could inhibit the activity of PPO; and that Cu^2+^ had a double effect on PPO, increasing the PPO activity at low concentrations and inhibiting the PPO activity at high concentrations. The inhibitor experiment showed that the inhibitory effects of EDTA and kojic acid were weak and that ascorbic acid and sodium pyrophosphate had good inhibitory effects. The purification and characterization of *Acetes chinensis* serve as guidelines for the prediction of enzyme behavior, leading to effective prevention of enzymatic browning during processing.

## 1. Introduction

Polyphenol oxidase (PPO) is an oxidase with Cu^2+^ in its active center [1]. It is widely present in microorganisms, plants, and animals. Phenolic substances act as respiratory transmission media, enabling the conversion of phenolic substances to achieve a state of dynamic balance [2,3]. When the tissue is damaged to a certain extent, the massive intervention of oxygen makes the conversion of phenolic substances unbalanced; the quinone in the tissue may cause accumulation; and the quinone substances are further transformed into melanin substances, which leads to the melanization of *Acetes chinensis*. As a result, the sensory perception of the product deteriorates and cannot be sold. The unit PPO oxidizes the colorless phenol of *Acetes chinensis* to colorless bisphenol in the presence of oxygen, and then, the binary PPO can change the colorless bisphenol to colored quinone [4], which is easily combined with amino acids in *Acetes chinensis* to form a brown complex commonly known as black spots [5,6,7].

The characteristics of PPO in aquatic products vary greatly. Zamorano et al. pointed out that the molecular weight of PPO in *Parapenaeus longirosteis* was 500 kDa, the optimal pH was 4.5, and the optimal temperature was 30 °C [8]. Sritunyalucksana and Cerenius pointed out that the molecular weight of PPO in *Penaeus monodon* was 78 kDa [9]. Benjakul et al. studied *Penaeus japonicas* and found that the molecular weight of PPO in *Penaeus japonicas* was 160 kDa, that the optimal pH was 8.76, and that the optimal temperature was 40–60 °C [10]. At present, there are few studies on PPO in *Acetes chinensis*. Therefore, it is necessary to study the characteristics of PPO in *Acetes chinensis*.

In this study, *Acetes chinensis* PPO was purified in a four-step procedure involving ammonium phosphate-buffered saline (PBS) treated, sulphate precipitation, DEAE anion-exchange chromatography, and Phenyl-Sepharose HP chromatography. By determining the optimal pH, optimal temperature and molecular weight of purified PPO, and the effects of different metal ions and different inhibitors on PPO, we can understand the characteristics of *Acetes chinensis* PPO. The purification and characterization of *Acetes chinensis* PPO may be helpful for the research on *Acetes chinensis* in processing and production.

## 2. Materials and Methods

### 2.1. Materials and Chemicals

*Acetes chinensis* used in this study was provided by Ruian Huasheng Aquatic Products Co., Ltd. (Wenzhou, China), which was harvested from the East China Sea. The body length was about 10–15 mm. Fresh samples were immediately subjected to the extraction of PPO. The other samples were stored in −80 °C ultra-low temperature refrigerator before use.

### 2.2. Purification of Acetes chinensis PPO

The crude enzyme solution was prepared using the method of Nirmal and Benjakul [11]. The fresh samples (250 g) were exactly weighed and dispensed into a clean glass beaker containing 500 mL of pH 6.8 phosphate buffer. The samples were vortex mixed (3 min), treated ultrasonically for 20 min, and vortex mixed (3 min) again. The extraction processes were repeated twice. High-speed (1000 r/min), low-temperature (4 °C) centrifugation was performed for 15 min, and the supernatant was obtained. The supernatant was used as a crude enzyme solution. The supernatant was subjected to fractionated ammonium sulfate precipitation for enzyme purification. The precipitated protein was dialysed with a 10 kDa dialysis membrane (HiMedia, Mumbai, India) by changing the dialysis buffer after every 4 h for 12 h. The samples were washed with PBS buffer, and the absorbance was measured at 595 nm every 2 min until the absorbance was lower than 0.05. After extensive washing of the column with the equilibration buffer, the protein was eluted by a stepwise change in the NaCl concentration to 1 mol/L in the same buffer. The PPO activity containing protein was eluted with the same buffer at 0.5 mL/min and collected at 4.0 mL per fraction, and ammonium sulfate (final concentration, 1.0 M) was added before DEAE-Cellulose chromatography. The sample containing 1.0 M ammonium sulfate was loaded onto the Phenyl-Sepharose HP column (1.5 × 20 cm), previously equilibrated with the above buffer containing 1.0 M ammonium sulfate. The column was washed with the same solution, and the protein was eluted with a linear gradient of 1.0 to 0 M ammonium sulfate in buffer.

### 2.3. Determination of Acetes chinensis PPO Activity

Referring to Ashida and Dohke’s method and adjusting it slightly [12], L-dopa was used as the specific substrate to measure the activity of PPO. For specific measurements, 100 μL of the enzyme solution was mixed with 100 μL of L-DOPA (0.015 mol/L), which was then reacted in a constant temperature water bath at 40 °C for 40 min followed by 2.8 mL of precooled purified water for reaction termination. The absorbance at 490 nm at the beginning and end of the reaction was measured with a spectrophotometer. The absorbance at 490 nm at the beginning and end of the reaction was measured with a spectrophotometer. Enzyme activity unit (U) was defined as an increase in absorbance of 0.001 per 1 min.

$$\mathrm{Enzyme}\;\mathrm{activity}\;=\frac{\Delta A490\;\mathrm{nm}}{40\;\min\;\times \;0.1\;\mathrm{mL}\;\mathrm{enzyme}\;\mathrm{solution}}$$

$$\mathrm{Relative}\;\mathrm{activity}=\frac{\mathrm{Activity}\;\mathrm{of}\;\mathrm{treated}\;\mathrm{Acetes}\;\mathrm{chinensis}\;\mathrm{PPO}}{\mathrm{Activity}\;\mathrm{of}\;\mathrm{untreated}\;\mathrm{Acetes}\;\mathrm{chinensis}\;\mathrm{PPO}}\;\times \;100\%$$

### 2.4. SDS-PAGE Gel Electrophoresis Determination

The protein content in the PPO solution was measured using the Coomassie Brilliant Blue method, and then, the concentration was adjusted to 1 mg/mL. In the SDS-PAGE analysis, the concentration of the concentrated gel was 5%, and the concentration of the separating gel was 8%. The sample solution was 0.1 mol/L pH 7.5. Tris-HCl buffer containing 2% SDS, 10% glycerol, and 0.02% bromophenol blue. The sample was heated in a boiling water bath for 5 min and then cooled in an ice water bath, the sample volume was 16 μL, and the voltage of the concentrated gel was at 80 V. Then, the sample was added to the separation gel.

The gels were electrophoresed at 200 V at room temperature until the bromophenol blue ran to the bottom of the gel. Lastly, the gel was removed and stained with Coomassie Brilliant Blue (0.1% Coomassie Brilliant Blue R-250, 45% methanol, and 10% glacial acetic acid) on a shaker for 30 min. After staining, the mixture was decolorized with Coomassie brilliant blue decolorizing solution (10% methanol and 10% glacial acetic acid) on a shaker to clear the strip and finally subjected to electrophoretic imaging scanning analysis.

### 2.5. Enzyme Kinetic Curve of Acetes chinensis PPO

L-DOPA was used as diphenolic substrates, and phosphate buffer (0.05 mol/L, pH = 7.2) was used to prepare L-DOPA solutions of different concentrations. The activity of PPO was measured using the method in Section 2.3, and the most suitable concentration was determined. According to Lineweaver–Burk’s method, the Michaelis constant (K_m_) and the maximum reaction velocity (V_m__ax_) rate were obtained.

### 2.6. Optimal pH of Acetes chinensis PPO Activity

The optimal pH was determined by dissolving L-Dopa in different pH buffers at 40 °C. After mixing 50 μL of purified PPO with 500 μL of L-Dopa buffers at different pH, the solution was incubated at 40 °C for 10 min to measure the activity of PPO. The pH buffers were sodium acetate buffer (pH 4.0–6.0), sodium phosphate buffer (pH 6.5–7.5), Tris-HCl buffer (pH 8.0–9.0), and Na_2_CO_3_-NaHCO_3_ buffer (pH 9.5–10.5).

### 2.7. Optimal Temperature of Acetes chinensis PPO Activity

To determine the optimal temperature of the enzyme reaction, the enzyme activity of PPO was measured at a temperature ranging from 25 to 85 °C. Phosphate buffer (0.05 mol/L, pH = 7.2) was used to ensure that the reaction system was at the optimal pH, and each reaction solution was reacted for 40 min in water bath. The activity of PPO was measured according to the method in Section 2.3 to study the best PPO temperature reflex.

### 2.8. Effect of Metal Ions on Acetes chinensis PPO Activity

The effects of metal ions (BaCl_2_, MnCl_2_, CuCl_2_, KCl, and CaCl_2_) on enzyme activity were tested using L-dopa as a substrate. Three different concentrations of metal ions (5 mmol/L, 10 mmol/L, and 15 mmol/L) were added to the reaction mixture, and the residual enzyme activity was measured after 30 min at 37 °C. The enzyme activity of the control mixture without metal ions was taken as 100% and then compared with the other treatments.

### 2.9. Effect of Inhibitor on Acetes chinensis PPO Activity

The enzyme solution was placed in phosphate buffer with a pH of 7.2, different inhibitors (sodium pyrophosphate, kojic acid, tea polyphenols, ascorbic acid, and EDTA) were added to the corresponding concentration (5 mmol/L, 10 mmol/L, and 15 mmol/L), and the enzyme activity was measured after 30 min at 37 °C. The enzyme activity of the control mixture without inhibitors was taken as 100% and then compared with the other treatments.

### 2.10. Statistical Analysis

All of the different treatments were repeated three times. The data were analyzed by the statistical software SPSS 26 for variance analysis. The values were expressed as mean ± SD and plotted with Origin 2019 software (2019, Microcal, Westborough, MA, USA).

## 3. Results and Discussion

### 3.1. Purification of Polyphenol Oxidase from Acetes chinensis

*Acetes chinensis* PPO was purified in a four-step procedure involving phosphate-buffered saline (PBS) treatment, ammonium sulphate precipitation, DEAE-Cellulose chromatography, and Phenyl-Sepharose HP chromatography. The enzyme liquid volume, total protein content, PPO enzyme activity, and purification multiple were changed accordingly. After the four steps of purification, the total volume of the extract decreased, with separation and purification from 1000 mL to 1 mL; the specific activity of the purified enzyme was increased from 21.2 U/mg to 643.4 U/mg, which was a 30.35 times increase in purification; and the recovery rate was 17.9% (Table 1). With the improvement in purification degree, the other enzymes gradually decreased and the concentration of PPO gradually increased, so the specific activity of PPO was greatly improved.

**Figure 1:**
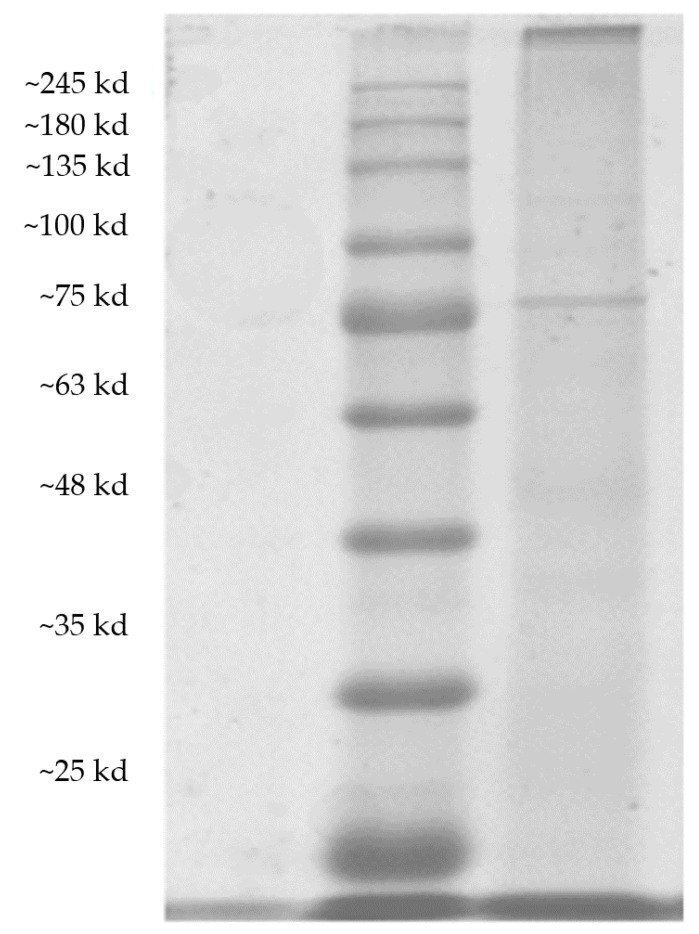
Analysis of the *Acetes chinensis* PPO electrophoresis.

As shown in Figure 1, it can be seen that there was only one band of purified PPO, which proves that the purification effect was very good. The band was compared with the marker to obtain a molecular weight of about 75 kDa [13]. The band represented the molecular weight of PPO protein in the *Acetes chinensis*. The molecular weight of PPO in cuttlefish was about 50 kDa [14]. The molecular weight of PPO in *Acetes chinensis* is similar to that in the human body.

### 3.2. Enzyme Kinetic Curve of the Acetes chinensis PPO Activity

The difference in the concentration of the substrate speeds up the enzyme reaction, which is different from the determination of PPO activity, which has a certain degree of influence. When studying the effects of temperature, pH, metal ions, etc. on enzyme activity, it is necessary to ensure that other conditions do not affect the enzyme activity, so we explored the optimal substrate concentration and Michaelis constant.

**Figure 2:**
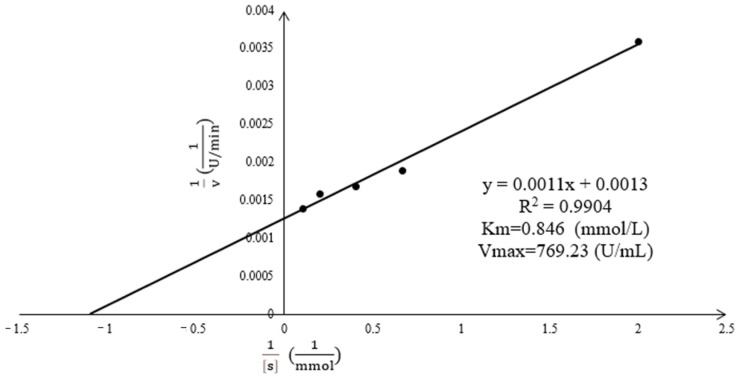
Lineweaver–Burk plots for the substrate of pyrocatechol on *Acetes chinensis* PPO.

The Michaelis equation of the *Acetes chinensis* PPO is shown in Figure 2. The kinetic parameters (K_m_ and V_max_) were determined from the Lineweaver–Burk plot (1/[S] versus 1/v) using the equation 1/v = 1/V_max_ + K_m_/V_max_[S], where v is the velocity of the reaction and [S] is the concentration of the substrate. With L-Dopa as the substrate, K_m_ = 0.846 mmol/L, which was lower than the K_m_ value (1.85 mmol/L) obtained by the phenoloxidase purified by Zamorano et al. [8] in the blood cells of *Penaeus vannamei*. The enzyme has a greater affinity for the substrate and reacts.

### 3.3. Effect of Temperature and pH on the Acetes chinensis PPO Activity

**Figure 3:**
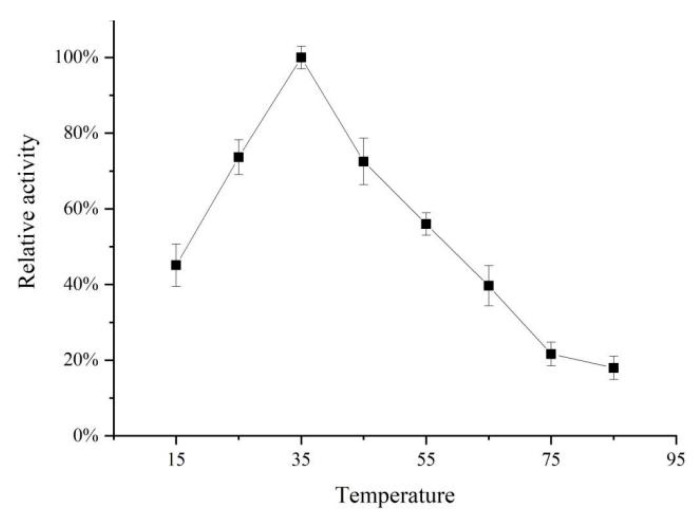
Optimal temperature of the *Acetes chinensis* PPO.

As shown in Figure 3, the *Acetes chinensis* PPO activity increased to a maximum at 35 °C and PPO had thermal stability at 25 °C to 45 °C. When the temperature was lower than 25 °C, the enzyme activity was inhibited and the reaction speed slowed down. When the temperature exceeded 55 °C, protein was denatured, resulting in a change in enzyme structure and a decrease in enzyme activity.

**Figure 4:**
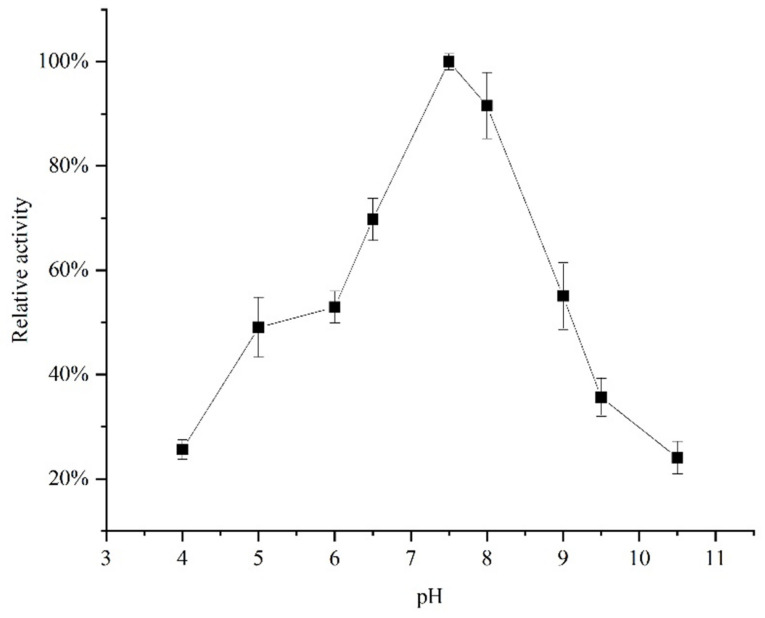
Optimal pH of the *Acetes chinensis* PPO.

Figure 4 showed that the specific enzyme activity of *Acetes chinensis* PPO reached its maximum near pH 7.5. When pH was lower than 6 or higher than 8, the specific activity of PPO decreased significantly. Han et al. showed that the optimal pH environment of *Penaeus vannamei* was around 7.5 [15]. The activity of *Acetes chinensis* PPO is inhibited by a too high or too low pH. According to a similar principle, the optimal extraction pH of *Acetes chinensis* PPO is about 7.5.

### 3.4. Effect of Metal Ions on the Acetes chinensis PPO Activity

**Figure 5:**
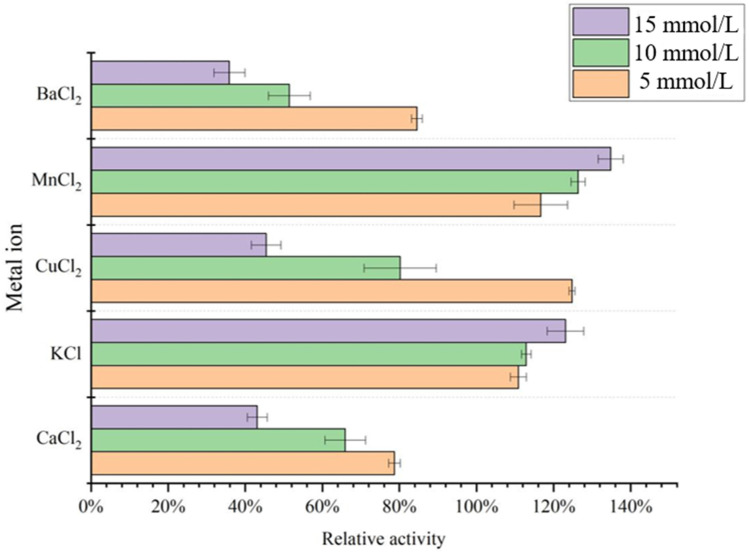
Effect of metal ions on the enzyme activity of *Acetes chinensis* PPO.

Metal ions play important roles in maintaining substrate binding in the active site of metalloenzymes and in controlling the redox activity of metalloenzymes in enzymatic reaction [3], and metal ions can reversibly change its valence to regulate the enzymatic reaction. The influence of metal ions on the activity of PPO was more complicated, and the degree of influence of different sources was different [16]. As shown in Figure 5, different concentrations of Ca^2+^ and Ba^2+^ inhibited the activity of PPO to varying degrees, and K^+^ and Mn^2+^ promoted the reaction and increased enzyme activity of PPO. Liu et al. purified PPO from the flower buds of Lonicera japonica and then found that Mn^2+^ promoted PPO and was an enzyme activator [17]; their result was similar to ours. When the Cu^2+^ concentration was low, the enzyme activity increased, and as the Cu^2+^ concentration increased, the activity of PPO gradually decreased. It may be due to the low concentration that the Cu^2+^ contained in the enzyme itself was separated and purified after adding a small amount of Cu^2+^; the affinity of the enzyme and the substrate was enhanced, thereby increasing the enzyme activity. However, the increase in Cu^2+^ concentration lead to a decrease in enzyme activity of PPO. The main reason for this was that the combination of Cu^2+^ and PPO reduced the affinity of the enzyme and the substrate or lead to a change in pH, leading to the inactivation of PPO [18].

### 3.5. Effect of Inhibitors on Acetes chinensis PPO Activity

**Figure 6:**
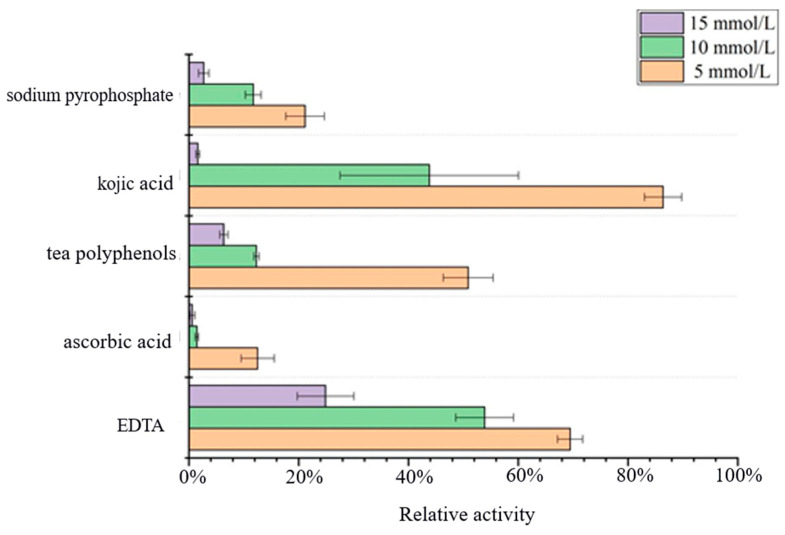
Effect of Inhibitors on the enzyme activity of *Acetes chinensis* PPO.

As shown in Figure 6, the activity of PPO was inhibited by ascorbic acid and sodium pyrophosphate even at low concentrations: 5 mmol/L of ascorbic acid lost 87.5% of the activity of PPO. López-Caballero et al. also yielded similar results [19]. The possible reason for this was that the neutral activity of PPO has two copper ions; due to the presence of the inhibitor, the Cu^2+^ in the active center was chelated, which destroys the binding site of the enzyme and the substrate, resulting in inactivation of the enzyme.

The inhibitory effect of EDTA, tea polyphenols, and kojic acid on the enzyme activity of PPO was not particularly obvious at low concentrations, but the enzyme activity also decreased significantly when the concentration increased. This may be because they can combine with quinone as an enzymatic reaction intermediate to form a stable colorless compound, thereby preventing the formation of brown pigments and reducing the production of colored substances. Therefore, their inhibitory effects were related to concentration [20].

## 4. Conclusions

In the present study, when L-dopa was used as the substrate, the enzymatic reactions catalyzed by PPO conform to the Michaelis equation: the maximum reaction velocity (V_max_) was 769.23 U/mL, and the Michaelis constant K_m_ was 0.846 mmol/L. It was found that the highest activity of PPO isolated and purified from the *Acetes chinensis* was at pH 7.5 and 35 °C. In the experiment of metal ions, it was found that medium and high concentrations of Ba^2+^, Cu^2+^, and Ca^2+^ had inhibitory effects on PPO but that medium and high concentrations of Mn^2+^ and K^+^ and low concentrations of Cu^2+^ had a certain promoting effect. In inhibitor studies, the inhibitory effects of EDTA and kojic acid were weak, and ascorbic acid and sodium pyrophosphate had good inhibitory effects. Nowadays, *Acetes chinensis* are gaining more and more attention all over the world, but the enzymatic browning during its storage period greatly reduce the quality of consumption. Understanding an effective mechanism for inhibiting the activity of PPO can inhibit enzymatic browning and is of great guiding significance.

## Figures and Tables

**Table 1:** Purification of PPO from *Acetes chinensis*.

Purification Stages	Volume (mL)	Total Activity (U)	Total Protein (mg)	Specific Activity (U/mg)	Yield (%)	Purification Fold
crude extract:0.05 M phosphate buffer	1000 ^a^ ± 0	7532 ^a^ ± 756	355 ^a^ ± 65.11	21.2 ^d^ ± 4.5	100 ^a^ ± 0	1 ^c^ ± 0
20–80% (NH4)_2_SO_4_ precipitation	248 ^b^ ± 16	4128 ^b^ ± 364	75 ^b^ ± 3.21	54.4 ^c^ ± 7.4	54.8 ^b^ ± 0.58	2.56 ^c^ ± 1.54
anion exchange:DEAE Sephacel column	20 ^c^ ± 7	1814 ^c^ ± 214	12 ^c^ ± 0.24	150.4 ^b^ ± 21.7	24.1 ^c^ ± 0.35	7.09 ^b^ ± 4.62
hydrophobic interaction:phynyl-Sepharose HP column	1 ^d^ ± 0.5	1351 ^c^ ± 133	2.1 ^d^ ± 0.04	643.4 ^a^ ± 65.2	17.9 ^c^ ± 0.12	30.35 ^a^ ± 14.30

Values are presented as means ± standard deviation; different number superscripts indicate the significant difference (*p* < 0.05) between each treatment in a respective column, use a, b, c, d to distinguish.

## Data Availability

The data presented in this study are available on request from the corresponding author.

## Sample Availability

Samples of the compounds are available from the authors.
